# Redefining chimeric antigen receptor T-cell (CAR-T) regulation: China’s responses to address secondary cancer risks of CAR-T therapy

**DOI:** 10.1186/s13045-024-01602-0

**Published:** 2024-10-08

**Authors:** Ruirong Tan, Rui Li, Meng-Yuan Dai, Miao Liu, Junning Zhao

**Affiliations:** 1 Translational Chinese Medicine Key Laboratory of Sichuan, Sichuan Institute for Translational Chinese Medicine, Sichuan Academy of Chinese Medicine Sciences, Chengdu, China; 2 Department of Radiation Oncology, Sichuan Cancer Hospital and Institute, Sichuan Cancer Center, Affiliated Cancer Hospital of University of Electronic Science and Technology of China, Chengdu, China; 3 Department of Gynecological Oncology, Zhongnan Hospital of Wuhan University, Wuhan, China; 4 Department of Pathology, Brigham and Women’s Hospital, Harvard Medical School, Boston, USA; 5 National Key Laboratory of Drug Regulatory Science, National Medical Products Administration (NMPA), Beijing, China

**Keywords:** CAR-T therapy, Secondary cancers, Drug regulation

## Abstract

Since the United States Food and Drug Administration (FDA) approved the first chimeric antigen receptor T-cell (CAR-T) therapy in 2017, it has marked a major breakthrough in cancer treatment, leading to a surge in global research and applications in this field. In recent years, China has made rapid progress, quickly catching up through heavy investment in CAR-T construction, preparation processes, and treatment strategies. China’s CAR-T therapy market is driven by substantial pharmaceutical investment targeting its vast population, yet high therapy costs remain uncovered by basic medical insurance. In November 2023, FDA issued a warning about the risk of secondary cancers in patients undergoing CAR-T therapy, sparking global concern. In fact, the China National Medical Products Administration (NMPA) preemptively implemented a series of measures to address the safety concerns of CAR-T therapy, emphasizing the risk of secondary cancers and advising lifelong monitoring as part of the approval process for CAR-T products. Nevertheless, additional regulatory measures are needed to address emerging risks, particularly the threat of secondary cancers. The authors believe that raising the standards for Investigational New Drug (IND) approval and establishing a dynamic reporting and feedback system based on real-world data will strengthen regulatory oversight and support the sustainable growth of the CAR-T industry in China.

To the Editor

Since the United States Food and Drug Administration (FDA) approved the first Chimeric Antigen Receptor T-cell (CAR-T) therapy in 2017, China has quickly caught up, becoming a major center for research and development in this growing field (Tables 1 and 2). China’s impressive advancement in this field is fueled by strong financial investments, high patient demand, and the distinct characteristics of its healthcare system. At the heart of this expansion are the Chinese government’s proactive policies and regulations, including fast-track drug approvals, financial incentives, and the development of essential infrastructure, such as biopharmaceutical industrial parks and cell therapy research centers [1]. According to the latest data, China has conducted over 850 CAR-T clinical trials, surpassing the 601 trials in the United States, highlighting its prominent role in CAR-T research and development (Table 1).

**Table 1:** Chimeric antigen receptor T-cell products developed in China

Product name	Generic name	Manufacturer	Target	Antigen-recognition domain	Approved indication	Approved by	Time of approval	Relevant studies
Zevor-cel	Zevorcabtagene Autoleucel	CARsgen Therapeutics, Shanghai, China	BCMA	a fully human BCMA-specific scFv (25C2)	Adult r/r MM progressed after at least 3 lines of systemic therapy, including a PI and an IMiD	NMPA	2024-02-23	[4]
CNCT19 (Inati-cel)	Inaticabtagene Autoleucel	Juventas Cell Therapy, Tianjin, China	CD19	a unique CD19 scFv derived from CD19 monoclonal antibody (HI19a)	Adult r/r B-ALL	NMPA	2023-11-07	[5]
Fucaso (Eque-cel)	Equecabtagene Autoleucel	IASO Bio, Nanjing, China	BCMA	a fully human scFv	Adult r/r MM progressed after at least 3 lines of systemic therapy, including a PI and an IMiD	NMPA	2023-06-30	[6]
Carvykti (Cilta-cel)	Ciltacabtagene Autoleucel	Janssen, New Jersey, US; Legend Biotech, Nanjing, China	BCMA	two llama (camelid) heavy chains (VH) as a scFv	Adult r/r MM after at least 4 lines of therapy, including a PI, an IMiD, and an anti-CD38 monoclonal antibody	FDA	2022-02-28	[7]
Relma-cel	Relmacabtagene Autoleucel	JW Therapeutics, Shanghai, China	CD19	scFv derived from a murine CD19-specific mAb (FMC63)	Adult r/r LBCL after at least 2 lines of systemic therapy	NMPA	2021-09-01	[8, 9]
Yescarta (Axi-cel)	Axicabtagene Ciloleucel	Fosun Kite Biotechnology, Shanghai, China	CD19	scFv derived from a murine CD19-specific mAb (FMC63)	Adult r/r LBCL after at least 2 lines of systemic therapy; adult LBCL fail to respond to first-line immunochemotherapy or relapse within 12 months after first-line immunochemotherapy	NMPA	2021-06-22	[10–12]

BCMA, B cell maturation antigen; scFv, single chain variable fragment; VH, variable region of the heavy chain; r/r MM, relapsed and/or refractory multiple myeloma; PI, proteasome inhibitor; IMiD, immunomodulatory drug; r/r B-ALL, relapsed and/or refractory B-cell acute lymphoblastic leukemia; r/r LBCL, relapsed and/or refractory large B-cell lymphoma; LBCL, large B-cell lymphoma; NMPA, National Medical Products Administration; FDA, Food and Drug Administration

**Table 2:** Comparative analysis of CAR-T therapies in the United States and China

Aspects	United States	China	Remarks
Year of first CAR-T approval	2017	2021	The US has a longer history in CAR-T development and played a pioneering role in the approval of these therapies.
Approved CAR-T therapies	6 products: Kymriah, Yescarta, Tecartus, Breyanzi, Abecma, Carvykti	5 products: Zevor-cel, CNCT19, Fucaso, Relma-cel, Yescarta	Yescarta is the only therapy approved in both countries. The US maintains leadership with more approved therapies, while China, despite starting later, has rapidly advanced with strong local innovation.
Targets	CD19, BCMA	CD19, BCMA	Both countries focus on the same key targets
Indications	MM, ALL, NHL, MCL, FL	MM, ALL, LBCL	The US covers a broader range of indications. China has more focused applications but is gradually expanding.
Number of clinical trials	601 (ClinicalTrials.gov)^@^	854 (ClinicalTrials.gov) + 247 (Chinese Clinical Trial Registry)^&^	China has now surpassed the US in the number of clinical trials, indicating a rapid expansion and growing leadership in CAR T-cell therapy research.
Future prospects and challenges	1. Innovation leadership: To maintain global leadership in CAR T-cell therapies; 2. Challenge: Facing global competition, affordability, and access issues.	1. Rapid growth: Driven by strong innovation and government support; 2. Challenge: Aligning regulatory and healthcare integration with the rapid expansion.	Both China and the US are at the forefront of CAR T-cell therapy development, each facing unique challenges that will influence the future of global healthcare innovation.

^@^According to ClinicalTrials.gov database, accessed on 15 August 2024

^&^China’s data combines two sources: 854 trials from ClinicalTrials.gov and 247 trials from the Chinese Clinical Trial Registry (both accessed on 15 August 2024). Some trials may be registered on both platforms, so overlap should be considered

US, United States; BCMA, B cell maturation antigen; MM, multiple myeloma; ALL, acute lymphoblastic leukemia; NHL, non-Hodgkin lymphoma; MCL, mantle cell lymphoma; FL, follicular lymphoma; LBCL, large B-cell lymphoma

In November 2023, the FDA issued a warning that the risk of T-cell malignancies is applicable to all approved BCMA-directed and CD19-directed genetically modified autologous CAR-T therapies, raising significant concerns about the safety of these treatments. The FDA officials reported receiving at least 22 cases of T-cell malignancies as adverse events in patients within two years after receiving CAR-T therapies [2]. However, due to the unknown characteristics and clinical status of the reported patients, FDA officials have stated that a definitive causal link between CAR-T therapy and secondary cancers has not been established [2]. A study of 449 CAR-T patients found a 3.6% incidence of secondary malignancies, with a projected 5-year incidence of 15.2% for solid tumors, 2.3% for hematologic malignancies, and a low risk of T-cell lymphoma [3]. In response to these concerns, in early 2024, the FDA required all six commercial CAR-T therapies to update their labels, adding secondary T-cell malignancies to the boxed warning section. Additionally, the FDA recommends that patients and clinical trial participants treated with these therapies undergo lifelong monitoring for new malignancies.

In recent years, the China NMPA has already implemented a series of regulatory measures to ensure the safety and efficacy of CAR-T therapy: (1) When approving the CAR-T products, the NMPA emphasized the potential risk of secondary malignancies, requiring the product labeling to highlight this risk and advising lifelong monitoring; (2) The NMPA requires that the production of all CAR-T products strictly adhere to Good Manufacturing Practice (GMP) standards to ensure quality control; (3) CAR-T clinical trials must be conducted at qualified institutions with long-term follow-up to monitor short- and long-term adverse reactions; (4) After the CAR-T products enter the market, the NMPA continuously monitors adverse reactions and takes necessary actions based on risk assessments; (5) The NMPA emphasizes that doctors and patients must be informed of potential risks, ensuring patients receive full risk information through detailed guidance and monitoring. Together, these measures establish a comprehensive regulatory framework that covers production, clinical application, and long-term follow-up, effectively safeguarding patient safety.

Although current policies and regulations in China have helped ensure the safety and efficacy of CAR-T therapy, they may not fully address emerging risks, including secondary cancers. Specifically, regarding the risk of secondary cancers, we believe it is necessary to implement the following measures to further strengthen regulation: (1) Establish a systematic risk management plan requiring strengthened adverse event reporting, with a focus on early identification and reporting of secondary cancers; (2) Raise entry standards for IND approval by requiring comprehensive preclinical data, GMP compliance, detailed safety monitoring, long-term follow-up plans, optimized trial designs, and stricter risk assessments, including monitoring for secondary cancers; (3) Enhance clinical supervision through regular quality inspections and long-term follow-ups; (4) Promote the collection and use of real-world data to effectively monitor and manage treatment outcomes and adverse reactions; (5) Establish a feedback mechanism to dynamically adjust regulations and promptly update clinical guidelines and product labels for emerging risks; (6) Enhance global regulatory consistency through international cooperation and information sharing. These measures are crucial for fortifying regulatory frameworks and proactively safeguarding against emerging risks, thereby ensuring the long-term safety and efficacy of CAR-T therapies on a global scale.

## Data Availability

No datasets were generated or analysed during the current study.
